# Data for sound pressure level prediction in lightweight constructions caused by structure-borne sound sources and their uncertainties

**DOI:** 10.1016/j.dib.2023.109292

**Published:** 2023-06-05

**Authors:** Albert Vogel, Joerg Arnold, Conrad Voelker, Oliver Kornadt

**Affiliations:** a Department of Building Physics, Bauhaus-Universität Weimar, 99423 Weimar, Germany; b Department of Building Physics/Energy-Efficient Buildings, Rheinland-Pfälzische Technische Universität Kaiserslautern-Landau, 67663 Kaiserslautern, Germany

**Keywords:** Building acoustics, Structure-borne sound, Sound pressure level prediction, Structure-borne sound sources, Comparison of measurement and prediction

## Abstract

When predicting sound pressure levels induced by structure-borne sound sources and describing the sound propagation path through the building structure as exactly as possible, it is necessary to characterize the vibration behavior of the structure-borne sound sources. In this investigation, the characterization of structure-borne sound sources was performed using the two-stage method (TSM) described in EN 15657. Four different structure-borne sound sources were characterized and subsequently installed in a lightweight test stand. The resulting sound pressure levels in an adjacent receiving room were measured. In the second step, sound pressure levels were predicted according to EN 12354-5 based on the parameters of the structure-borne sound sources. Subsequently, the predicted and the measured sound pressure levels were compared to obtain reliable statements on the achievable accuracy when using source quantities determined by TSM with this prediction method.

In addition to the co-submitted article (Vogel et al., 2023), the sound pressure level prediction according to EN 12354-5 in detail is described. Furthermore, all data used are provided.

**Specifications Table**

**Table:** 

Subject	Civil and Structural Engineering, building acoustic
Specific subject area	Sound pressure level prediction, structure-borne sound sources, lightweight constructions, uncertainties
Type of data	Tables; images
How the data were acquired	The data were measured by microphones and acceleration meters. For measuring plate mobilities an electrodynamic shaker was used to excite the reception plates. For the characterization of the structure-borne sound sources, only surface velocities on reception plates were measured. For those measurements a laser Doppler Vibrometer was also used.
Data format	Analyzed
Description of data collection	The data file “Data for calculation.xlsx” contains all numerical values necessary for the calculation of the sound pressure level caused by the structure-borne sound sources. The manuscript also contains a detailed sketch of the building elements considered. The provided values were measured in small frequency bands (Δf = 1 Hz) as well as third-octave bands. To determine the source parameters, the raw data were measured exclusively in small frequency bands to calculate the source parameters from these values. Subsequently, the calculated source parameters were converted into third-octave band values (see Table 1 in the article and the data file). The description of the individual data is given at the top of each table in the manuscript.
Data source location	All data are available at Bauhaus-Universität Weimar Chair of building physics Coudraystrasse 11a 99423 Weimar
Data accessibility	Repository name: Mendeley Data Identification number: 10.17632/sn39mbyngb.1 Direct link to data: https://data.mendeley.com/datasets/sn39mbyngb
Related research article	This data article supports the following research article: A. Vogel, J. Arnold, C. Voelker, O. Kornadt, Applicability of the structure-borne sound source characterization two-stage method as well as the parameters derived in sound pressure level predictions in lightweight constructions. Applied Acoustics, 205 (2023) https://doi.org/10.1016/j.apacoust.2023.109242.

## Value of the Data

• The calculation and dataset presented in this article allow other researchers, especially acousticians, to conduct further calculations to reduce the uncertainties of the prediction method. For example: using frequency depending on radiation efficiency as well as new information concerning the sound propagation in buildings, and simulation of the investigated setup.
• This full dataset of a sound pressure level prediction provides also detailed information about the structure especially the walls in the test stand
• This full dataset of a sound pressure level prediction caused by structure-borne sound sources provides detailed information about the characterized values of the structure-borne sound sources
• This dataset illustrates the difference between predicted and measured uncertainties to specific frequencies as well as to single values representing the whole frequency range (total sum, arithmetic mean, A-weighted sum levels, etc.)

## 1 Objective

The supported article [1] presents analysis, discussions, and insights into the data and measurement method of the two-stage method (TSM) while characterizing a shaker, compressor, extractor fan, and ventilation unit (typical structure-borne sound sources). To determine the uncertainties of the predicted sound pressure levels based on these source parameters, subsequently the sound pressure levels were measured in a lightweight test stand by mounting the sound sources on a flanking wall and compared with predicted data. This article presents the full dataset of these sound pressure level predictions due to the four structure-borne sound sources including the measured data of the source characterization with TSM and all necessary data.

## 2 Data Description

In [6] the full dataset used for the sound pressure level prediction is provided. The data consist of numerical values and related formulas, which are necessary for the sound pressure level prediction in rooms due to structure-borne sound sources. The data also characterize the building elements of a lightweight test stand and the vibrational behavior of the sources used.

**Fig. 1:**
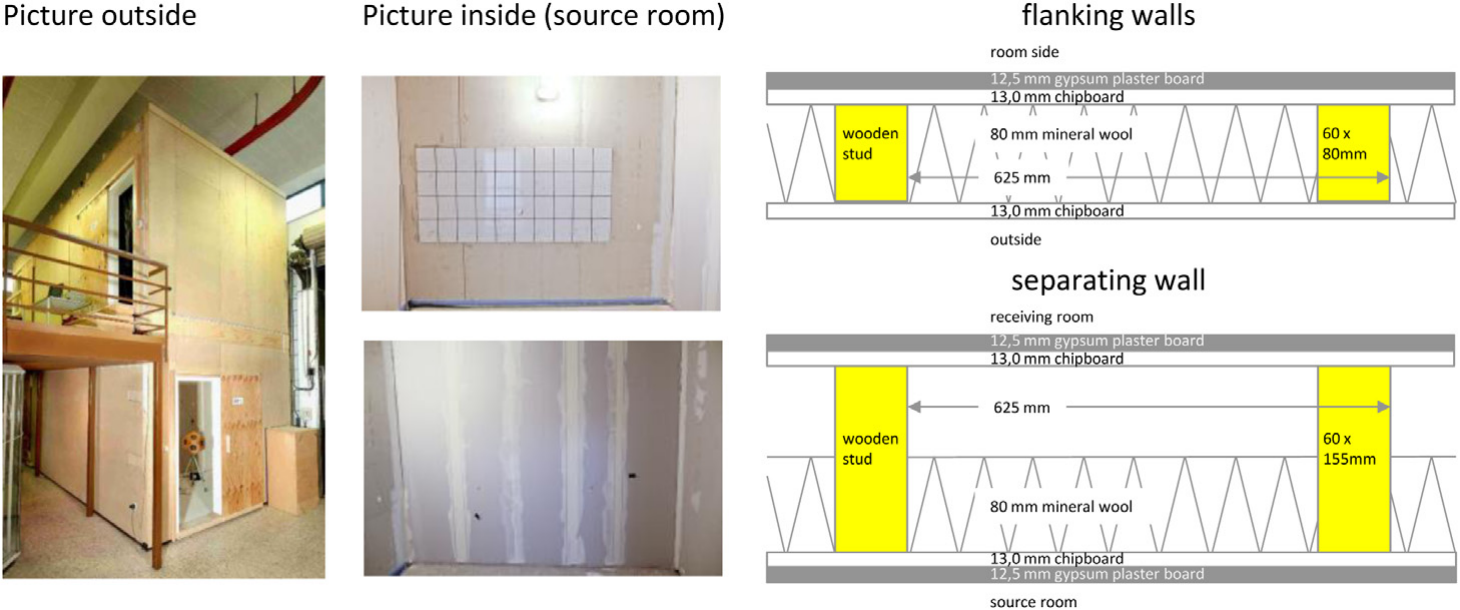
Left - lightweight test stand [3]; middle, top - flanking wall with tiled section in source room; middle, bottom - separating wall in source room; right top - construction of the flanking walls; right bottom - construction of the separating wall.

Fig. 1 shows the lightweight test stand, sketches and dimensions, where the measurement of the data was done. Table 1 shows the characteristic structure-borne sound source parameters *v*_f_, *F*_b_, and *Y*_s_. Table 2 shows constant parameters and room dimensions. Table 3 shows the receiving mobility *Y*_r_ and Table 4 the resulting coupling term *D*_C,i_ for each source. Table 5 shows the adjustment term *D*_as,i_ and installed structure-borne sound power *L*_Ws,inst,i_. Table 6 provides the sound reduction index *R*_i_ of the walls. Table 7 contains the structural reverberation time *T*_s,i_ of the walls. shows the equivalent absorption length *a*_i_ Table 8. Table 9 contains the direction-averaged junction velocity level difference $\bar{\frac{D_{v,\text{ij}}+D_{v,\text{ji}}}{2}}$. Table 10 provides the reverberation time *T*_60_ and equivalent absorption area *A* of the source and receiving rooms. Table 11 contains the vibration reduction indices *K*_ij_; the flanking sound reduction index *R*_ij_ and the flanking sound reduction coefficient *R*_ij,ref_. Table 12 provides the sound pressure levels *L*_n,s,ij_ for paths 1 and 2 and the resulting sum *L*_n,s_ in the receiving room, predicted and measured values. Table 13 shows the differences between the predicted and measured normalized sound pressure levels *L*_n,s_ in the receiving room as mean values across all investigated sources. Table 14 shows the list of measurement equipment.

**Table 1:** Characteristic structure-borne sound parameters of the sources used, measured with two-stage method according to [2].

	Compressor			Shaker		
Frequency third-octave band [Hz]	Free velocity *v*_f [m/s]_	blocked force *F*_b_ [N]	source mobility *Y*_s_ [s/kg]	Free velocity *v*_f [m/s]_	blocked force *F*_b_ [N]	source mobility *Y*_s_ [s/kg]
50	6.7 E-3	7.8 E-2	8.6 E-2	4.2 E-3	8.1 E-1	5.2 E-3
63	1.8 E-3	5.8 E-2	3.0 E-2	3.9 E-3	7.7 E-1	5.1 E-3
80	5.2 E-3	5.7 E-1	9.1 E-3	3.8 E-3	9.1 E-1	4.2 E-3
100	3.3 E-3	4.0 E-1	8.3 E-3	3.5 E-3	7.9 E-1	4.4 E-3
125	2.2 E-3	4.7 E-1	4.8 E-3	3.1 E-3	8.5 E-1	3.7 E-3
160	2.0 E-3	7.8 E-1	2.6 E-3	2.6 E-3	9.2 E-1	2.8 E-3
200	1.7 E-3	1.6 E+0	1.1 E-3	2.2 E-3	9.1 E-1	2.4 E-3
250	2.0 E-3	2.3 E+0	8.6 E-4	1.8 E-3	9.3 E-1	1.9 E-3
315	3.1 E-3	2.9 E+0	1.1 E-3	1.5 E-3	1.0 E+0	1.5 E-3
400	3.2 E-3	2.9 E+0	1.1 E-3	1.4 E-3	1.2 E+0	1.1 E-3
500	5.6 E-3	6.5 E+0	8.6 E-4	1.1 E-3	1.2 E+0	9.7 E-4
630	6.1 E-3	4.7 E+0	1.3 E-3	9.9 E-4	1.4 E+0	7.1 E-4
800	3.6 E-3	2.5 E+0	1.4 E-3	8.7 E-4	1.4 E+0	6.1 E-4
1000	2.8 E-3	2.5 E+0	1.1 E-3	7.4 E-4	1.4 E+0	5.4 E-4
1250	1.9 E-3	1.5 E+0	1.3 E-3	6.5 E-4	1.3 E+0	4.8 E-4
1600	1.8 E-3	9.4 E-1	1.9 E-3	6.2 E-4	1.4 E+0	4.4 E-4
2000	1.7 E-3	6.0 E-1	2.8 E-3	5.4 E-4	1.2 E+0	4.3 E-4
2500	1.3 E-3	3.3 E-1	3.9 E-3	5.0 E-4	1.3 E+0	4.0 E-4
3150	4.3 E-4	1.9 E-1	2.3 E-3	4.9 E-4	1.0 E+0	4.9 E-4
4000	3.7 E-4	2.1 E-1	1.8 E-3	5.5 E-4	9.9 E-1	5.5 E-4
5000	2.9 E-4	2.5 E-1	1.2 E-3	9.0 E-4	1.3 E+0	6.9 E-4

	Ventilation unit			Extractor fan		
Frequency third-octave band[Hz]	Free velocity *v*_f [m/s]_	blocked force *F*_b_ [N]	source mobility *Y*_s_ [s/kg]	Free velocity *v*_f [m/s]_	blocked force *F*_b_ [N]	source mobility *Y*_s_ [s/kg]

50	8.7 E-4	4.2 E-1	2.1 E-3	6.7 E-3	3.0 E-1	2.2 E-2
63	1.0 E-4	2.0 E-1	5.3 E-4	1.4 E-3	4.7 E-1	2.9 E-3
80	2.7 E-4	3.0 E-1	9.0 E-4	2.1 E-3	7.1 E-1	3.0 E-3
100	6.6 E-4	1.4 E-1	4.6 E-3	5.7 E-4	7.8 E-1	7.4 E-4
125	3.9 E-4	1.3 E-1	3.0 E-3	7.9 E-4	3.1 E-1	2.5 E-3
160	2.5 E-4	3.1 E-1	8.3 E-4	7.6 E-4	4.8 E-1	1.6 E-3
200	2.0 E-4	4.3 E-1	4.8 E-4	4.3 E-4	3.7 E-1	1.2 E-3
250	5.4 E-4	3.2 E-1	1.7 E-3	3.6 E-4	2.6 E-1	1.4 E-3
315	2.7 E-4	1.4 E-1	1.9 E-3	2.3 E-4	2.2 E-1	1.0 E-3
400	1.7 E-4	1.1 E-1	1.6 E-3	1.8 E-4	2.0 E-1	9.0 E-4
500	1.0 E-4	7.9 E-2	1.3 E-3	2.0 E-4	1.2 E-1	1.6 E-3
630	7.5 E-5	8.6 E-2	8.8 E-4	1.1 E-4	1.4 E-1	8.1 E-4
800	1.6 E-4	1.1 E-1	1.5 E-3	1.1 E-4	6.6 E-2	1.7 E-3
1000	6.2 E-5	8.2 E-2	7.5 E-4	8.5 E-5	3.4 E-2	2.5 E-3
1250	5.6 E-5	8.6 E-2	6.6 E-4	5.3 E-5	3.2 E-2	1.6 E-3
1600	3.8 E-5	1.2 E-1	3.3 E-4	5.1 E-5	2.1 E-2	2.4 E-3
2000	1.0 E-4	1.3 E-1	8.2 E-4	3.6 E-5	1.9 E-2	1.9 E-3
2500	1.9 E-5	1.2 E-1	1.5 E-4	3.7 E-5	1.6 E-2	2.3 E-3
3150	1.7 E-5	1.4 E-1	1.2 E-4	2.9 E-5	1.2 E-2	2.5 E-3
4000	1.0 E-5	1.7 E-1	5.8 E-5	3.1 E-5	7.2 E-3	4.3 E-3
5000	5.9 E-6	2.1 E-1	2.8 E-5	2.8 E-5	4.6 E-3	6.2 E-3

**Table 2:** Room parameters and constants.

geometry		source room (sr)	receiving room (rr)
depth [m]		2.95	2.95
width [m]		2.89	3.67
height [m]		2.55	2.55
			
volume [m³]		21.74	27.61
area walls [m²]	flank sr	flank rr	separating (sep.) wall rr
	7.37	9.36	7.52
Common length of the joints *l*_ij_ [m]	flank sr – flank rr	flank sr – sep. wall rr	
	2.55	2.55	
radiation efficiency *σ* (50-5000 Hz)	1	[-]	
sound velocity in air c_0_	342	[m/s]	
area mass double-leafed wall	24	[kg/m²]	
*ρ*_0_ ∙ *c*_0_ (air)	400	[Ns/m³]

**Table 3:** Mobility (absolute and real part) of the flanking wall sr, where the source is mounted; mean over 3 coupling points.

third-octave band [Hz]	|*Y*i| [m/Ns]	Re{*Y*i}[m/Ns]
50	1.1 E-4	1.9 E-4
63	1.7 E-4	2.2 E-4
80	1.9 E-4	2.9 E-4
100	2.4 E-4	3.6 E-4
125	2.7 E-4	3.7 E-4
160	3.0 E-4	4.7 E-4
200	3.9 E-4	5.3 E-4
250	4.1 E-4	5.3 E-4
315	4.3 E-4	5.6 E-4
400	5.0 E-4	6.5 E-4
500	5.2 E-4	6.5 E-4
630	5.8 E-4	7.5 E-4
800	6.5 E-4	8.6 E-4
1000	7.4 E-4	9.2 E-4
1250	7.2E-4	8.6 E-4
1600	7.7 E-4	9.3 E-4
2000	9.1 E-4	1.1 E-3
2500	9.1 E-4	1.1 E-3
3150	1.0 E-3	1.3 E-3
4000	1.4 E-3	1.7 E-3
5000	1.7 E-3	2.0 E-3

**Table 4:** Coupling term *D*_C,i_ of the flanking wall sr, where the source is mounted.

third-octave band	Compressor	Shaker	Ventilation unit	Extractor fan
[Hz]	[dB]	[dB]	[dB]	[dB]
50	26.6	14.6	10.9	20.7
63	21.4	13.9	6.2	11.6
80	15.2	12.1	6.7	10.7
100	13.9	11.4	11.6	5.6
125	11.6	10.6	9.8	9.2
160	8.4	8.7	5.2	6.8
200	5.8	7.8	4.7	5.9
250	5.5	7.3	6.9	6.4
315	5.7	6.5	7.1	5.6
400	5.5	5.6	6.2	5.3
500	5.3	5.4	5.9	6.3
630	5.6	5.0	5.1	5.0
800	5.5	4.8	5.6	5.8
1000	5.3	5.2	5.1	6.6
1250	5.6	5.4	5.3	6.0
1600	6.0	5.5	5.9	6.5
2000	6.5	5.8	5.2	5.8
2500	7.4	6.0	8.3	6.2
3150	5.8	5.7	9.3	5.9
4000	5.2	6.0	13.1	6.5
5000	5.3	6.0	16.9	6.9

**Table 5:** Adjustment term *D*_as,i_; installed structure-borne sound power lever *L*_Ws,inst,i_ on the flanking wall in the source room.

*L*_Ws,inst_					

third-octave band	*D*_as,i_	compressor	shaker	Ventilation unit	Extractor fan

[Hz]	[dB]	[dB]	[dB]	[dB]	[dB]
50	-23.6	60.6	80.8	80.1	72.4
63	-15.6	58.8	80.9	66.9	76.5
80	-8.2	79.5	83.3	72.4	81.0
100	-17.3	77.2	83.1	68.1	80.9
125	-23.7	78.6	83.6	67.4	74.7
160	-22.7	83.6	85.1	73.7	78.9
200	-25.4	88.6	85.1	74.7	76.1
250	-29.0	91.1	85.0	75.5	73.4
315	-30.0	93.7	85.4	68.6	71.5
400	-28.9	94.1	86.6	66.6	70.4
500	-31.6	100.3	85.6	63.2	67.6
630	-34.5	99.0	86.4	63.0	67.0
800	-35.0	93.9	86.1	66.7	62.9
1000	-37.7	93.2	84.9	62.0	57.9
1250	-37.0	89.0	84.0	61.6	56.3
1600	-37.0	86.2	84.0	60.5	53.9
2000	-38.4	83.5	82.5	66.0	52.5
2500	-39.1	78.8	82.0	55.5	51.4
3150	-30.2	73.4	81.2	54.2	49.4
4000	-30.7	73.7	81.4	49.2	47.0
5000	-38.0	73.3	84.8	44.0	44.2

**Table 6:** Sound reduction index *R*i in dB.

third-octave band	Flanking wall sr *R*_f_	Flanking wall rr *R*_f_	Separating wall *R*_D_
[Hz]	[dB]	[dB]	[dB]
50	23.3	23.3	23.3
63	18.3	18.3	18.3
80	13.4	13.4	13.4
100	22.3	22.3	22.3
125	30.6	30.6	30.6
160	30.4	30.4	30.4
200	33.4	33.4	33.4
250	36.7	36.7	36.7
315	38.0	38.0	38.0
400	37.7	37.7	37.7
500	40.4	40.4	40.4
630	44.1	44.1	44.1
800	44.6	44.6	44.6
1000	46.1	46.1	46.1
1250	46.6	46.6	46.6
1600	49.2	49.2	49.2
2000	51.6	51.6	51.6
2500	49.3	49.3	49.3
3150	46.4	46.4	46.4
4000	49.9	49.9	49.9
5000	54.2	54.2	54.2

**Table 7:** Structural reverberation time *T*_s,i_ of the walls.

third-octave band	Flanking wall sr	Flanking wall rr	separating wall rr
[Hz]	[s]	[s]	[s]
50	0.88	0.72	0.51
63	0.45	0.4	0.51
80	0.25	0.31	0.35
100	0.26	0.19	0.28
125	0.17	0.16	0.22
160	0.14	0.12	0.13
200	0.13	0.12	0.12
250	0.14	0.13	0.15
315	0.13	0.08	0.09
400	0.11	0.08	0.1
500	0.11	0.09	0.1
630	0.09	0.07	0.08
800	0.09	0.07	0.09
1000	0.12	0.08	0.08
1250	0.09	0.07	0.11
1600	0.05	0.08	0.14
2000	0.04	0.09	0.32
2500	0.08	0.11	0.3
3150	0.02	0.17	0.55
4000	0.01	0.08	0.51
5000	0.02	0.07	0.35

**Table 8:** Equivalent absorption length *a*_i_.

Third-octave band	Flanking wall sr	Flanking wall rr	Separating wall rr
[Hz]	[m]	[m]	[m]
50	2.4	3.7	4.2
63	4.1	5.9	3.7
80	6.6	6.8	4.8
100	5.7	9.9	5.4
125	7.8	10.5	6.1
160	8.4	12.4	9.2
200	8.0	11.1	8.9
250	6.7	9.1	6.4
315	6.4	13.2	9.5
400	6.7	11.7	7.5
500	6.0	9.3	6.8
630	6.6	10.7	7.5
800	5.8	9.5	5.9
1000	3.9	7.4	6.0
1250	4.7	7.6	3.9
1600	7.4	5.9	2.7
2000	8.3	4.7	1.1
2500	3.7	3.4	1.0
3150	13.2	2.0	0.5
4000	23,4	3,7	0,5
5000	10,5	3,8	0,6

**Table 9:** Direction-averaged junction velocity level difference $\bar{\frac{D_{v,\text{ij}}+D_{v,\text{ji}}}{2}}$ (mean of 3 shaker positions).

third-octave band	flank sr - flank rr	flank sr - sep. wall rr
[Hz]	[dB]	[dB]
50	22.4	19.1
63	19.9	15.1
80	15.3	18.8
100	15.7	19.9
125	23.7	20.1
160	24.2	22.7
200	21.6	22.7
250	20.2	20.6
315	22.6	24.9
400	24.7	27.1
500	26.0	27.2
630	23.2	27.1
800	24.7	28.5
1000	21.5	26.2
1250	22.2	27.6
1600	21.6	27.3
2000	17.5	26.9
2500	18.4	27.6
3150	18.8	27.2
4000	19.0	28.9
5000	14.8	27.6

**Table 10:** Reverberation time *T*_60_ and equivalent absorption area *A* of source and receiving room.

third-octave band	*T*_60_ sr	*T*_60_ rr	*A* sr	*A* rr
[Hz]	[s]	[s]	[m²]	[m²]
50	0.82	1.17	4.25	3.78
63	1.18	0.65	2.95	6.81
80	0.42	0.55	8.30	8.05
100	0.61	0.45	5.71	9.84
125	0.79	0.86	4.41	5.15
160	0.72	0.80	4.84	5.53
200	0.81	0.93	4.30	4.76
250	0.88	0.96	3.96	4.61
315	0.81	1.01	4.30	4.38
400	0.94	1.13	3.71	3.92
500	1.08	1.11	3.23	3.99
630	1.02	1.20	3.42	3.69
800	1.06	1.16	3.29	3.82
1000	0.99	1.15	3.52	3.85
1250	0.92	1.05	3.79	4.22
1600	0.85	1.01	4.10	4.38
2000	0.87	0.99	4.01	4.47
2500	0.82	0.93	4.25	4.76
3150	0.79	0.90	4.41	4.92
4000	0.80	0.90	4.36	4.92
5000	0.76	0.86	4.59	5.15

**Table 11:** Vibration reduction indices *K*_ij_; flanking sound reduction index *R*_ij_; flanking sound reduction coefficient *R*_ij,ref_.

	Vibration reduction indices		flanking sound reduction index		flanking sound reduction coefficient	
third-octave band	*K*_Ff_	*K*_Fd_	*R*_Ff_	*R*_Fd_	*R*_Ff,ref_	*R*_Fd,ref_
[Hz]	[dB]	[dB]	[dB]	[dB]	[dB]	[dB]
50	21.8	18.2	49.7	46.2	50.9	47.4
63	17.0	13.2	39.9	36.2	41.1	37.4
80	11.1	15.3	29.1	33.4	30.3	34.6
100	11.0	16.5	37.9	43.5	39.1	44.8
125	18.2	15.8	53.5	51.1	54.7	52.3
160	18.2	17.4	53.2	52.5	54.5	53.7
200	15.9	17.5	53.9	55.6	55.1	56.9
250	15.3	16.6	56.6	58.0	57.8	59.2
315	17.0	20.1	59.6	62.7	60.9	64.0
400	19.3	22.7	61.6	65.1	62.8	66.3
500	21.3	23.2	66.3	68.3	67.6	69.6
630	18.1	22.7	66.8	71.5	68.0	72.7
800	20.0	24.9	69.2	74.2	70.5	75.4
1000	18.3	23.4	69.0	74.2	70.2	75.5
1250	18.5	25.4	69.7	76.6	70.9	77.9
1600	17.5	24.9	71.3	78.8	72.5	80.0
2000	13.6	26.2	69.8	82.5	71.1	83.8
2500	16.9	28.8	70.8	82.8	72.1	84.0
3150	15.8	27.2	66.8	78.3	68.1	79.5
4000	13.3	27.7	67.8	82.3	69.1	83.6
5000	10.9	27.6	69.7	86.5	70.9	87.8

**Table 12:** Sound pressure levels *L*_n,s,ij_ for paths Ff and Fd and the resulting sum *L*_n,s_ in the receiving room, predicted and measured values.

	Compressor					
third-octave band	1 path Ff	2 path Fd	3 sum	4 sum	5 measured	6 measured

[Hz]	[dB]	[dB]	[dB]	[dB(A)]	[dB]	[dB(A)]
50	30.6	34.1	35.7	5.5	35.6	5.3
63	30.6	34.3	35.9	9.7	27.1	0.8
80	54.7	50.4	56.1	33.6	34.4	12.0
100	52.7	47.1	53.8	34.7	31.8	12.6
125	44.9	47.3	49.3	33.2	39.0	22.8
160	49.2	50.0	52.6	39.2	47.6	34.3
200	56.1	54.4	58.4	47.5	54.0	43.1
250	59.6	58.2	62.0	53.4	52.9	44.2
315	60.2	57.1	61.9	55.3	51.5	44.9
400	57.6	54.1	59.2	54.4	51.8	47.0
500	61.8	59.8	63.9	60.7	54.2	51.0
630	62.8	58.1	64.1	62.2	51.9	50.0
800	55.7	50.8	57.0	56.2	47.2	46.4
1000	58.0	52.8	59.2	59.2	45.0	45.0
1250	52.3	45.4	53.1	53.7	42.2	42.8
1600	48.0	40.6	48.7	49.7	35.9	36.9
2000	48.2	35.5	48.4	49.6	30.2	31.4
2500	43.2	31.2	43.5	44.8	30.9	32.1
3150	32.9	21.4	33.2	34.4	32.4	33.6
4000	32.7	18.2	32.8	33.8	34.0	34.9
5000	37.7	20.9	37.8	38.3	27.1	27.7

	Shaker					
third-octave band	path Ff	path Fd	sum	sum	measured	measured

[Hz]	[dB]	[dB]	[dB]	[dB(A)]	[dB]	[dB(A)]
50	50.7	54.2	55.8	25.6	51.3	21.1
63	52.7	56.5	58.0	31.8	59.8	33.5
80	58.5	54.2	59.9	37.4	49.7	27.4
100	58.5	52.9	59.6	40.5	51.5	32.4
125	50.0	52.4	54.3	38.2	54.0	37.8
160	50.7	51.4	54.1	40.7	50.5	37.3
200	52.7	51.0	54.9	44.0	54.0	43.1
250	53.5	52.1	55.8	47.2	51.0	42.3
315	51.8	48.7	53.5	46.9	53.5	46.9
400	50.0	46.6	51.7	46.9	54.5	49.7
500	47.0	45.0	49.2	46.0	55.5	52.2
630	50.2	45.5	51.5	49.6	56.5	54.6
800	47.9	43.0	49.1	48.3	56.3	55.5
1000	49.7	44.5	50.8	50.8	54.1	54.1
1250	47.4	40.4	48.2	48.8	52.8	53.4
1600	45.8	38.3	46.5	47.5	48.2	49.2
2000	47.2	34.5	47.4	48.6	49.6	50.8
2500	46.4	34.4	46.7	48.0	54.1	55.3
3150	40.7	29.3	41.0	42.2	57.2	58.4
4000	40.4	25.9	40.5	41.5	53.9	54.9
5000	49.2	32.4	49.3	49.8	56.7	57.2
	Ventilation unit					
third-octave band	path Ff	path Fd	sum	sum	measured	measured

[Hz]	[dB]	[dB]	[dB]	[dB(A)]	[dB]	[dB(A)]
50	50.1	53.6	55.2	25.0	37.2	6.9
63	38.7	42.5	44.0	17.8	38.7	12.4
80	47.6	43.3	49.0	26.5	34.2	11.8
100	43.6	37.9	44.6	25.5	39.4	20.2
125	33.8	36.1	38.1	22.0	38.1	21.9
160	39.3	40.0	42.7	29.3	37.0	23.7
200	42.3	40.5	44.5	33.6	32.9	22.1
250	44.0	42.6	46.4	37.8	36.1	27.4
315	35.0	31.9	36.8	30.2	46.9	40.2
400	30.0	26.6	31.6	26.8	39.2	34.4
500	24.6	22.6	26.7	23.5	26.7	23.4
630	26.8	22.1	28.0	26.1	23.5	21.5
800	28.6	23.6	29.8	29.0	25.3	24.5
1000	26.8	21.6	27.9	27.9	24.7	24.7
1250	24.9	18.0	25.7	26.3	19.6	20.2
1600	22.3	14.8	23.0	24.0	18.1	19.1
2000	30.7	18.0	30.9	32.1	16.1	17.3
2500	19.9	8.0	20.2	21.5	15.9	17.2
3150	13.7	2.2	14.0	15.2	15.8	17.0
4000	8.2	-6.3	8.3	9.3	11.4	12.4
5000	8.4	-8.4	8.5	9.0	10.1	10.7

	Extractor fan					
third-octave band	path Ff	path Fd	sum	sum	measured	measured

[Hz]	[dB]	[dB]	[dB]	[dB(A)]	[dB]	[dB(A)]
50	42.4	45.9	47.5	17.3	54.4	24.1
63	48.3	52.1	53.6	27.4	45.4	19.2
80	56.2	51.9	57.6	35.1	51.3	28.9
100	56.4	50.7	57.4	38.3	45.4	26.3
125	41.1	43.5	45.4	29.3	48.0	31.8
160	44.4	45.2	47.8	34.4	35.3	22.0
200	43.6	41.9	45.9	35.0	34.2	23.4
250	41.8	40.5	44.2	35.6	33.4	24.7
315	37.9	34.8	39.6	33.0	38.5	31.9
400	33.9	30.4	35.5	30.7	33.3	28.5
500	29.0	27.0	31.1	27.9	31.6	28.4
630	30.8	26.1	32.1	30.2	29.9	28.0
800	24.7	19.8	25.9	25.1	26.6	25.8
1000	22.7	17.5	23.9	23.9	24.8	24.8
1250	19.7	12.8	20.5	21.1	20.7	21.3
1600	15.7	8.2	16.4	17.4	18.1	19.0
2000	17.3	4.5	17.5	18.7	14.8	16.0
2500	15.8	3.9	16.1	17.4	16.9	18.2
3150	8.9	-2.6	9.2	10.4	18.2	19.4
4000	6.0	-8.5	6.1	7.1	16.8	17.7
5000	8.7	-8.2	8.8	9.3	15.7	16.2

## 3 Experimental Design, Materials and Methods

The data article presents the prediction method including all necessary data concerning the structure-borne sound sources (compressor, shaker, ventilation unit, and extractor fan) and the sound pressure level prediction. The structure-borne sound source characterization was done by the two-stage method, according to [2]. Therefore, the sources were mounted on a heavy and a light reception plate (approx. 3 - 5 m²) and were switched on. The induced structure-borne sound power was determined on the plate surfaces using the measured surface velocity. Using the two reception stages heavy and light one can make simplifications regarding the receiver mobility (very high or very low compared to the source mobility) and this yields to installation-independent source parameters. Detailed information about the structure-borne sound source characterization method itself is provided in [2]. In [1], the characterization of the sources used is described in detail. The determined source parameters free velocity *v*_f_, blocked force *F*_b_, and source mobility *Y*_S_ are provided in Table 1. All measured data used for the investigation of the sound pressure level prediction were measured in a lightweight test stand at Working Group 1.72 Applied Acoustics, PTB Braunschweig.

**Table 13:** Differences of the predicted and measured normalized sound pressure levels *L*_n,s_ in the receiving room; mean value across all investigated sources.

Frequency range	calculation of the mean	with shaker (Shaker; compressor; ventilation unit; extractor fan)		without shaker (compressor; ventilation unit; extractor fan)	
[Hz]		dB	dB(A)	dB	dB(A)
50 – 5000	single third-octave bands	6.8		7.3	
	total sum level	5.5	5.4	7.2	5.3
100 – 3150	single third-octave bands	6.4		**7.0**	
	total sum level	5.1	5.3	6.5	**5.2**
50 – 1000	single third-octave bands	6.8		7.7	
	total sum level	5.6	4.8	7.2	5.1

**Table 14:** List of measurement equipment used.

Sound analyzers	Oros: OR3-Serie 32-Channel Sinus: Soundbook 4-Channel Polytec: Controller OFV 5000
Scanning Vibrometer	Polytec: PSV-400-3D
Impedance heads, acceleration meters	PCB, Dytran: Sensitivities 100 mV/g; 500 mV/g; 1000 mV/g
Electrodynamic shaker	TIRA: 2 x TV 51110, power sine/noise: 100 N/70 N
Microphones	Microtech Gefell: MM210, sensitivity 50 mV/Pa

### 3.1 Data of the Characterized Structure-borne Sound Sources

Using to the measurement method described above Table 1 provides the measured installation independent source parameters.

### 3.2 Calculation of the Sound Pressure Levels According to EN 12354-5 in a Lightweight Test Stand

#### 3.2.1 Lightweight Test Stand at PTB Braunschweig

The lightweight test stand at the PTB in Braunschweig is a wooden plate construction with a length of 7.10 m and a width of 3.25 m. There are two adjacent rooms on each of the two floors with a room height of 2.55 m, so that sound transmission can be reproduced horizontally, vertically, and diagonally with a coupling of structure-borne sound sources to the partition wall or flanking elements.

The perimeter walls are made of 60-mm x 80-mm timber studs spaced 625 mm apart and filled with 80-mm mineral wool. On the outside, these flank walls are covered with 13-mm chipboard, and on the inside with 13-mm chipboard and 12.5-mm plasterboard. In the interior wall area of one of the flanking walls, on which the structure-borne sound sources were mounted for this investigation, there was also a partially tiled section of approx. 0.80 m x 2.00 m.

The substrate of the tiles (plasterboard) was first treated with deep primer before the tile adhesive was applied, so that it does not lose all of its moisture and thus its adhesive strength on the wall. Then the tiles were glued and grouted. The tiles are standard bathroom tiles with the dimensions 20 × 25 [cm] and a weight of approx. 750 g per tile.

Both separating walls, one per floor, consist of a 60-mm x 155-mm wooden framework that is also filled with 80-mm mineral wool. They are covered on both sides with 13-mm chipboard and 12.5-mm plasterboard. On both floors, the separating walls are butt-jointed to the flanking exterior walls (with continuous planking) and arranged offset to each other on each floor, so that all element connections of the lightweight test stand are always designed as T-joints (no cross-joints existing).

**Fig. 2:**
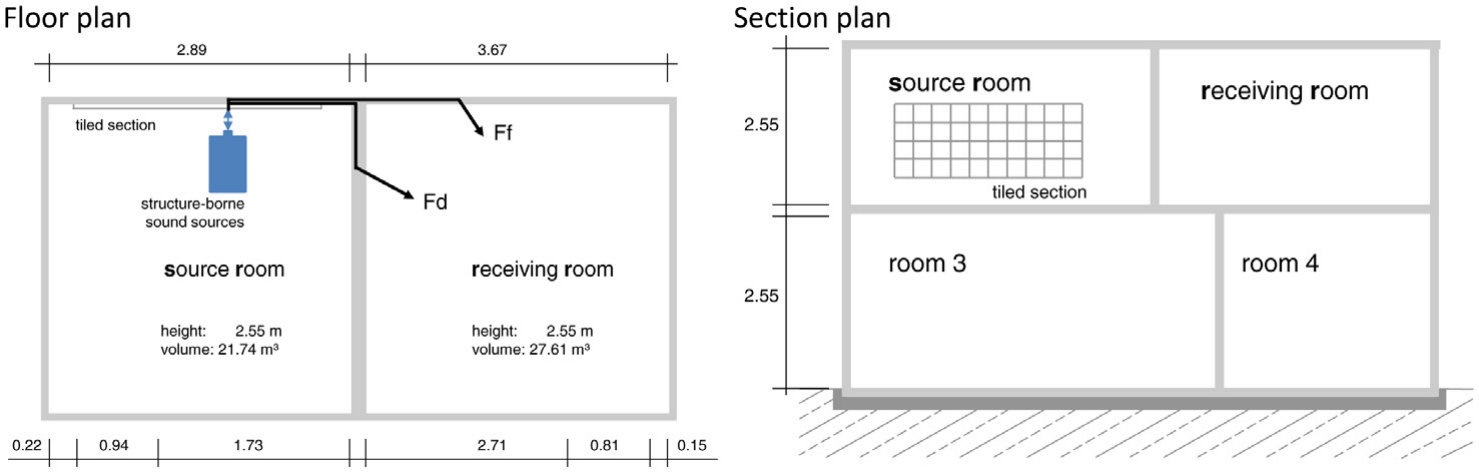
Left: Floor plan of the Lightweight test stand with relevant transmission paths: Fd – **F**lanking wall source room to **d**irect/ separating element, Ff – **F**lanking wall source room to **f**lanking wall receiving room; right: Section plan of the Lightweight test stand.

The upper ceiling, which closes off the test stand, is constructed in the same way as the surrounding perimeter walls. The bottom floor consists of a reinforced concrete floor slab on which 20-mm polystyrene, 10-mm wood fiber insulation, and 20-mm Fermacell gypsum fiber boards are laid from bottom to top. The separating ceiling is designed as a typical wooden beam ceiling with 180-mm high ceiling beams. On top, it is finished from bottom to top with a 22-mm flat pressed board, 30-mm mineral fill, a 10-mm wood fiber insulation board, and a 20-mm Fermacell gypsum fiberboard. Since the three investigated sound sources as well as the shaker were connected to a flanking exterior wall on the upper floor and the standardized sound pressure level was only investigated in the neighbor receiving room, the ceiling components were neglected in the prediction according to EN 12354-5. The relevant dimensions of the lightweight test stand and the element constructions are shown in Figs. 1 and 2. Here, the dimensions of the separating and flanking walls differ, because a higher sound reduction index of the separating wall was chosen.

#### 3.2.2 Prediction Method

The normalized sound pressure level $L_{n,s}$ in the receiving room induced by structure-borne sound sources is predicted with a prediction method according to [4,5]. The equations and the full data set for the prediction are given in this section.

**Table:** 

$L_{n,s}=10\mathrm{lg}\sum_{j=1}^{n}10^{L_{n,s,\text{ij}}/10}$	[dB]	1	
*L*_n,s_		resulting in normalized sound pressure level for the given transmission situation	[dB]
*L*_n,s,ij_		Normalized sound pressure level for the transmission path between source at element *i* and radiating element *j* in the receiving room	[dB]
$L_{n,s,ij}=L_{Ws,inst,i}-D_{sa,i}-R_{ij,ref}-101g\frac{S_{i}}{S_{ref}}-101g\frac{A_{ref}}{4}$	[dB]	2	
*L*_Ws,inst,i_		Installed structure-borne sound power level on the source element	[dB]
*D*_sa,i_		Adjustment term for the conversion of a structure-borne sound excitation into an airborne sound excitation of the source element *i*	[dB]
*R*_ij,ref_		Flanking sound reduction coefficient from element *i* in the source room to element *j* in the receiving room, related to an reference area *S*_ref_ = 10 m²	[dB]
*S*_i_		Geometric Area of element *i* with the installed source	[m²]
*S*_ref_		Reference *area S*_ref_ = 10 m²	
*A*_ref_		Equivalent reference absorption area *A*_ref_ = 10 m²	
$L_{\text{Ws},\text{inst},i}=L_{\text{Ws},c}-D_{c,i}$	[dB]	3	
*L*_Ws,inst,i_		Installed structure-borne sound power level on the source element	[dB]
*L*_Ws,c_		Characteristic structure-borne sound power level	[dB]
*D*_c,i_		Coupling term source - receiving structure	[dB]
$L_{Ws,c}=10.1g\frac{v_{f}^{2}}{W_{ref}}\frac{1}{|Y_{S}|}$	[dB]	4	
*v*_sf_		Free velocity of the source	[m/s]
*W*_ref_		Reference power 10^-12^ W	[W]
*Y*_S_		Mobility of the receiving structure	[m/Ns]
$D_{C,i}=101g\frac{{|Y_{s}|}^{2}+{|Y_{i}|}^{2}}{|Y_{s}|\text{Re}\left\{Y_{i}\right\}}$	[dB]	5	
*Y*_S_		Source mobility	[m/Ns]
*Y*_i_		Mobility of the receiving structure	[m/Ns]
$D_{sa,i}=101g\frac{2\pi m_{i}2,2\tau i}{\rho_{0}c_{o}T_{s,i}\sigma_{i}}$	[dB]	6	
*m*_i_		Area-related mass of element *i*, where the source is mounted	[kg/m²]
*τ*_i_		Transmission coefficient of element *i* for airborne sound	[-]
*ρ*_0_		Density of air at 20°C	[kg/m³]
*c*_0_		Speed of sound in air	[m/s]
*T*_s,i_		Structure-borne sound reverberation time of element *i*	[s]
*σ*_i_		Radiation efficiency of element *i*	[-]
*R*_i_		Sound reduction index of element *i*	[dB]
$\tau =10^{-\frac{R_{i}}{10}}$	[-]	7	
$R_{\text{ij}}=\frac{R_{i}}{2}+\Delta R_{i}+\frac{R_{j}}{2}+\Delta R_{j}+K_{\text{ij}}+10\cdot \mathrm{lg}\frac{S_{s}}{l_{0}l_{\text{ij}}}$	[dB]	8	
$R_{\text{ij},\text{ref}}=R_{\text{ij}}+10\cdot \mathrm{lg}\frac{S_{\text{ref}}}{S_{s}}$	[dB]	9	
*R*_ij_		Flanking sound reduction index	[dB]
*R*_ij,ref_		Flanking sound reduction coefficient, related to an reference area *S*_ref_ = 10 m²	
*R*_i_ ; *R*_j_		Sound reduction index of element *i* and element *j*	[dB]
Δ*R*_i_ ; Δ*R*_j_		Airborne sound improvement due to an additional facing shell	[dB]
*K*_ij_		Vibration reduction index	[dB]
*l*_ij_		Joint length	[m]
*l*_0_		Reference joint length; *l*_0_ = 1 m	[m]
*S*_S_		Geometric area of the separating wall	[m²]
*S*_0_		Reference area *S*_ref_ = 10 m²	[m²]

The sound reduction index of the flanking walls is taken from the measurement of the sound reduction index of the separating wall, the constructions are similar.

Since the existing joints were not sufficiently known, the vibration reduction indices *K*_ij_ were determined experimentally according to Equation 10 by measuring the velocity level differences *D*_v,ij,_ and *D*_v,ji_ (Table 9) for the relevant transmission paths. The equivalent absorption lengths *a*_i_ and *a*_j_ (Table 8) were calculated using Equation 11 and the measured structure-borne sound reverberation time *T*_s,i_.

**Table:** 

$K_{\text{ij}}=\frac{D_{v,\text{ij}}+D_{v,\text{ji}}}{2}+\left(10\cdot \mathrm{lg}\frac{l_{\text{ij}}}{\sqrt{a_{i}a_{j}}}\right)$	[dB]	10	
*K*_ij_		Vibration reduction indices	[dB]
*l*_ij_		Common length of the junction between element i and j	[m]
*a*_i_ and *a*_j_		Equivalent absorption length of the elements i and j	[m]
$a_{i}=\frac{2,2\pi^{2}\cdot S_{i}}{c_{0}\cdot T_{s,i}}\sqrt{\frac{f_{\text{ref}}}{f}}$	[dB]	11	
*S*_i_		Geometric area of element i	[m²]
*T*_s,i_		Structural reverberation time of the element i	[s]
*c*_0_		Speed of sound in air	[m/s]
*f*_ref_		Reference frequency *f*_ref_ = 100 Hz	[Hz]
*f*		Centre frequency of the one-third octave band under consideration	[Hz]

Table 11 presents the vibration reduction index, the flanking sound reduction index, and the flanking sound reduction coefficient for both transmission paths, Ff and Fd.

#### 3.2.3 Comparison of Predicted and Measured Sound Pressure Levels

Columns 1 and 2 of Table 12 contain the predicted normalized sound pressure level components of the individual transmission paths *L*_n,s,ij_. Columns 3 and 4 contain the energetic sum of columns 1 and 2 as normalized sound pressure level *L*_n,s_ in the receiving room. Columns 5 and 6 show the measured values of the normalized sound pressure level *L*_n,s_ in the receiving room.

Table 13 shows the differences between the predicted and the measured values of the normalized sound pressure levels. The values represent the energetic mean value across all investigated sources. Since the shaker is an ideal source of structure-borne sound for the characterization and prognosis method (punctiform one-point contact with the receiving structure), it cannot be regarded as a common source of structure-borne sound. Therefore, the deviations are shown with (columns 3 and 4) as well as without the shaker (columns 5 and 6). For the frequency range relevant to building acoustics in Germany (normative requirements of 100 – 3150 Hz), the A-weighted total level results in an average deviation of 5.2 dB, and the arithmetic mean value of all 16 single third-octave band differences is 7.0 dB. It must be discussed, which frequency range is valid and if the levels must be A-weighted because of the typical acting of structure-borne sound sources in the low and very low frequency range and because of their tonal behavior, which can be very disturbing.

### 3.3 Measurement Equipment

In Table 14, the main components of the measurement equipment are listed, which were used for the investigation of the characterization method, the characterization of the sources, and the sound pressure level measurements.

## Ethics Statements

No ethical issues are associated with this work.

## CRediT authorship contribution statement

**Albert Vogel:** Investigation, Writing – original draft. **Joerg Arnold:** Investigation, Validation. **Conrad Voelker:** Supervision. **Oliver Kornadt:** Supervision.

## Declaration of Competing Interests

The authors declare that they have no known competing financial interests or personal relationships that could have appeared to influence the work reported in this paper.

## Data Availability

Data for sound pressure level prediction (Original data) (Mendeley Data).
